# Evaluating the PANSS using item response theory in forensic psychiatric samples from five European nations

**DOI:** 10.1038/s41537-025-00668-0

**Published:** 2025-11-25

**Authors:** Andreas Wippel, Giovanni de Girolamo, Pawel Gosek, Janusz Heitzman, Laura Iozzino, Inga Markiewicz, Donato Martella, Marco Picchioni, Hans-Joachim Salize, Annemarie Unger, Johannes Wancata, Rainer W. Alexandrowicz

**Affiliations:** 1 Medical University of Vienna, Clinical Division of Social Psychiatry, Vienna, Austria; 2 IRCCS Istituto Centro San Giovanni di Dio Fatebenefratelli, Unit of Epidemiological Psychiatry and Digital Mental Health, Brescia, Italy; 3 Department of Psychiatry, Center of Postgraduate Medical Education, Warsaw, Poland; 4 Institute of Psychiatry and Neurology, Department of Forensic Psychiatry, Warsaw, Poland; 5 Department of Forensic and Neurodevelopmental Science, Institute of Psychiatry, Psychology and Neuroscience, King’s College London, London, UK; 6 St Magnus Hospital, Haslemere, Surrey, UK; 7 Central Institute of Mental Health Mannheim, Medical Faculty Mannheim/Heidelberg University, Heidelberg, Germany; 8 University of Klagenfurt, Institute of Psychology, Klagenfurt, Austria

**Keywords:** Schizophrenia, Human behaviour

## Abstract

Item Response Theory (IRT) describes a set of statistical models describing how individual items in a test or questionnaire relate to the underlying characteristic or trait that the test claims to measure. Until now IRT models have not been applied to the Positive and Negative Syndrome Scale (PANSS) in forensic and general psychiatric samples to establish its psychometric properties and explore the link between psychotic symptom severity and violent behavior in schizophrenia. This study investigated patients with schizophrenia spectrum disorders and a history of violence from forensic institutions and non-violent patients from general psychiatric settings in five European countries. A total of 398 participants were assessed using the PANSS. IRT analysis revealed a poor model fit for the Partial Credit Model (PCM) with considerably disordered thresholds for most items. Differential item functioning (DIF) revealed significant differences between the two groups, notably for items hypothetically linked to violence risk, such as delusions and hostility. These findings reveal potential limitations when trying to compare PANSS scores across these two clinical populations.

## Introduction

Individuals with schizophrenia spectrum disorders (SSDs) exhibit a significantly elevated risk of violent behavior, violent offending, and homicide compared to the general population^1,2^. This association is multifactorial and shaped by a constellation of clinical, historical, and contextual factors, including genetic and biological predispositions, male sex, younger age, history of physical/sexual abuse, victimization or criminal history, comorbid substance use, antisocial behavior, poor treatment adherence, unemployment, and greater overall symptom severity^3–9^.

The Positive and Negative Syndrome Scale (PANSS^10^) is a 30-item clinician-rated instrument used to assess the severity of current positive and negative symptoms of psychosis. It is widely employed in clinical trials and psychiatric research and is considered the gold standard for evaluating symptom severity in SSDs.

Understanding the relationship between symptom severity and violent behavior in schizophrenia has been a longstanding research focus. A meta-analysis by Witt et al.^9^, including 110 studies across 73 independent samples and encompassing a total of 45,533 individuals, demonstrated that higher positive symptom scores and elevated total PANSS scores were associated with more violent behavior. Similarly, Buizza et al.^11^, in their meta-analysis, found that although both forensic and non-forensic patients presented with low levels of psychopathology, forensic patients consistently scored higher across all PANSS subscales. Specific symptom profiles—particularly delusional beliefs involving perceived threats and the emotional impact of those symptoms—have been linked to an increased risk of violence^12^.

Two theoretical frameworks have been proposed to explain the link between psychotic symptoms and violence. The first, proposed by Link and Stueve^13^, highlighted the role of “Threat-Control-Override” (TCO) symptoms—defined as delusional beliefs that one’s thoughts or actions are controlled by external forces (control override), and that one is being persecuted or targeted (threat). These symptoms were found to be more frequently associated with violent behavior. Recent studies, however, suggested a more nuanced understanding of the impact of symptomatology, including TCO symptoms, on violent behavior in individuals with SSDs, underscoring the complexity of this association^11,12,14^. The second hypothesis concerns command hallucinations, i.e., auditory hallucinations in which individuals perceive voices as omnipotent or coercive. These hallucinations may lead to increased risk of violence, particularly when the individual feels compelled to act in response to the perceived authority of the voices^15^.

Despite these theoretical models, the relationship between various psychotic symptoms and violence remains ambiguous, partly due to the dynamic and fluctuating nature of symptoms across different phases of the disorder^16^. Moreover, the often-lengthy time gap between the commission of the violent act and the psychiatric assessment along with variations in diagnoses, mental state, and environmental factors makes it difficult to draw reliable conclusions regarding causality between positive psychotic symptoms and violence.

Despite those findings, given the centrality of symptoms to the risk of violence in clinical practice, it is crucial to examine how the PANSS functions psychometrically in forensic populations. To date, most studies investigating the psychometric properties of the PANSS have employed methods rooted in Classical Test Theory (CTT^17^). CTT approaches typically begin by computing total or subscale scores, followed by the application of various statistical techniques—such as reliability coefficients and factor analysis—many of which are based on item intercorrelations. However, CTT makes several assumptions, including the treatment of item scores as interval-scaled and adherence to certain axioms that are not empirically testable. Moreover, CTT methods depend on statistical assumptions (e.g., normality of distributions, unidimensionality) that are often violated in practice, particularly in clinical populations.

In contrast, Item Response Theory (IRT^18^) provides a more nuanced and empirically grounded approach: it focuses on the relationship between each individual item and the latent trait the scale (in this case, the PANSS) is intended to measure. A central advantage of IRT is that it treats item responses as ordered categorical variables, rather than assuming they are metric, as in CTT. This is especially appropriate for the types of ordinal items commonly used in psychiatric and psychological assessments. By modeling item characteristics at this granular level, IRT can identify issues and inconsistencies within specific response categories, offering a more detailed and robust evaluation of scale performance compared to CTT.

Although IRT has previously been applied to the PANSS, revealing its psychometric limitations and inconsistencies^19–21^, these investigations have been conducted solely in general adult psychiatric populations. Despite its use in forensic settings, no IRT-based psychometric analysis of the PANSS has been conducted in a forensic setting. This gap is important because differential characteristics of forensic samples may influence the performance of the scale, potentially leading to misestimation of symptom severity and misguided clinical decisions. The central aim of our study was to explore whether individual PANSS items function differently across forensic and non-forensic samples of individuals with a SSD—an approach known as Differential Item Functioning (DIF^22^). Thus, we tested the hypothesis that the polytomous Rasch model fits the data adequately and shows no differences between the two populations.

## Methods

### Sample

This study formed part of the European Study on Risk Factors for Violence in Mental Disorder and Forensic Care (EU-VIORMED^23^). EU-VIORMED is a collaborative research project that aims to improve the quality of forensic psychiatric care in Europe. The field work was conducted in five European countries: Austria, Germany, Italy, Poland, and the United Kingdom. Patients with a primary diagnosis of a DSM-V SSD^24^ and a history of significant interpersonal violence were recruited from several forensic psychiatric institutions in the five countries. Significant interpersonal violence was defined as having committed a homicide, attempted homicide or other assault that caused serious physical injury to another person. Diagnoses were made by the treating clinicians. All subjects were aged between 18 and 65 years. The main exclusion criteria were: (i) confirmed intellectual disability; (ii) traumatic brain injuries or organic brain disorders; (iii) not being able to speak the national language fluently. To compare with general psychiatric patients ("Controls”) we selected gender and age-matched patients with SSDs who had never committed an act of significant violence, from general psychiatric services. The study was approved by the relevant Ethics Committees of each participating site. All participants provided written informed consent before entering the study after a full verbal and written description of the study’s aims and methods.

### Assessments

All subjects were evaluated by research assistants employed by the study and centrally trained. Socio-demographic, core clinical, and criminological data were rated from patient interviews, cross-referenced with medical and criminal records and clinician review. Overall, 398 patients with an SSD (221 forensic and 177 non-forensic patients) were assessed using the PANSS^10^, based on a semi-structured patient interview and clinical observation. PANSS scoring used the original standard scoring model^10^; the PANSS overall total score ranges from 30 to 210. All research workers underwent official centralized PANSS training in 2018 provided by the PANSS Institute and were certified PANSS raters. Further details regarding design, assessments and sampling can be found elsewhere^23,25^.

The PANSS employs a seven-point ordered response format for each symptom—ranging from absent, minimal, mild, moderate, moderate severe, severe, to extreme—with detailed guidelines on how each symptom should be rated. The standard 30-symptom version of the PANSS consists of three subscales: Positive Symptoms (7 items), Negative Symptoms (7 items), and General Psychopathology (16 items), each evaluated by calculating the sum of symptom scores within the subscale. Cognitive functioning was assessed by using the Brief Assessment of Cognition in Schizophrenia (BACS)^26^.

### Analysis

We applied the Partial Credit Model (PCM; Masters, 1982), an IRT model specifically designed for ordered polytomous data. The PCM estimates both person parameters and threshold parameters for each item, with thresholds representing the points on the latent trait continuum that separate adjacent response categories. The proper ordering of these thresholds is a core assumption of the model, any disordering of thresholds constitutes a violation and may indicate that the response categories are not functioning as intended. A standard way to visualize PCM item functioning is through Category Characteristic Curves (CCCs), which depict the probability of endorsing each response category across the range of the latent trait. These curves provide a clear graphical representation of how well the response options discriminate across levels of symptom severity. One of the key advantages of the PCM is that person and item parameters are expressed on the same latent scale, allowing for direct comparison. Ideally, the person’s trait (here: severity) estimates should be well-aligned with the item thresholds—i.e., they should be located within the same range on the latent continuum. Misalignment suggests that the items may be psychometrically “too difficult” or “too easy” for the population under investigation. In this study, these relationships are illustrated using Person-Item Category Characteristic Curve (PIccc) diagrams, following the approach outlined by Kabic and Alexandrowicz^27^.

To check whether the PCM delivers an adequate representation of the data, we applied the M2 test (yielding the *χ*^2^ test-statistic, *df*, *p*-value, and the Normed *χ*^2^, i.e., NC = *χ*^2^/*df*). We further considered the root mean squared error of approximation (RMSEA) along with its confidence limits, the standardized root means squared residual (SRMSR), the Tucker-Lewis-Index (TLI), the comparative fit index (CFI), the Akaike information criterion (AIC), the Bayesian information criterion (BIC), the sample adjusted BIC (SABIC), and the Hannan-Quinn criterion (HQ). For the latter, values are considered acceptable between 2^28^ and 5^29^. A significance level of 5% was assumed for all statistical tests. As this is the first study to compare a forensic to a non-forensic sample of patients with an SSD, we did not use alpha adjustment.

The second focus of our analysis was on Differential Item Functioning (DIF)—that is, whether items perform equivalently across known subgroups beyond what would be expected by random variation. Specifically, we compared item parameter estimates between the forensic and non-forensic adult samples. Significant differences in these estimates suggest that individuals with the same level of the latent trait respond differently depending on group membership, which is a psychometric concern indicating potential item bias. DIF was assessed using a multi-group χ^2^-test. Importantly, this test assumes model fit under the null hypothesis; therefore, statistically significant results indicate model violation and are considered undesirable in this context.

The application of the PCM is particularly relevant because it justifies the use of simple, unweighted sum scores, as commonly applied in clinical settings. In other words, the PCM provides the necessary psychometric foundation for interpreting summed item scores as valid representations of a respondent’s symptom severity.

All analyses were conducted using R^30^. For the IRT analyses, we employed the mirt package^31^ and the PIccc diagrams were generated using the RMX package^27^.

## Results

### Socio-demographic, clinical and forensic characteristics

Of the 575 patients who expressed an interest in the study, 175 declined to take part, 99 forensic subjects, 30.9%, and 76 non-forensic, 30.0%). The final sample comprised 398 patients with a primary diagnosis of an SSD (Table 1): 221 forensic patients with a history of serious interpersonal violence and 177 general adult controls without a history of serious violence. The groups differed by country (*p* = 0.007) but were similar in age (*p* = 0.291). The majority of participants were male (84.4%), with a higher male proportion in the forensic group (*p* = 0.019). Clinical characteristics showed that schizophrenia (76.4%) and schizoaffective disorder (15.8%) were the most common diagnoses in both groups. Forensic patients had a younger age at first psychiatric contact, though both groups had a mean illness duration of over 13 years. Nearly all patients of both groups received psychotropic medication. The control group showed higher scores for verbal memory, verbal fluency, and symbol coding tasks according to BACS, while both groups did not differ for other cognitive tasks. Further details can be found in de Girolamo et al.^23^.

**Table 1:** Socio-Demographic Characteristics Of Forensic Patients With Ssd And Controls.

	Forensic group *N* = 221 *N* (%)	Control group *N* = 177 *N* (%)	*p*-value
**Country of recruitment**			
*Austria*	50 (22.6)	50 (28.2)	**0.007**
*Germany*	36 (16.3)	33 (18.6)	
*Italy*	39 (17.6)	36 (20.3)	
*Poland*	56 (25.3)	48 (27.1)	
*United Kingdom*	40 (18.1)	10 (5.6)	
**Sex**			
*Male*	195 (88.2)	141 (79.7)	**0.019**
*Female*	26 (11.8)	36 (20.3)	
**Ethnicity**			
*White*	191 (86.4)	165 (93.2)	0.089
*Other ethnic groups*	29 (13.2)	10 (5.6)	
*Don’t know/won’t say*	1 (0.5)	2 (1.1)	
**Age**			
*18-29*	50 (22.6)	52 (29.4)	0.291
*30-41*	93 (42.1)	60 (33.9)	
*42-53*	45 (20.4)	40 (22.6)	
*54-65*	33 (14.9)	25 (14.1)	
**Marital status**			
*Married or cohabiting*	10 (4.5)	15 (8.5)	0.223
*Single*	183 (82.8)	144 (81.4)	
*Divorced or widowed*	28 (12.7)	18 (10.2)	
**Education years**, Mean (SD)	11.5 (3.3)	12.9 (3.4)	**<0.001**
**Highest occupational status**			
*Never worked/ Student/housewife*	32 (14.5)	25 (14.3)	0.427
*Unskilled worker*	114 (51.6)	77 (44.0)	
*Skilled worker*	64 (29.0)	63 (36.0)	
*Professional*	11 (5.0)	10 (5.7)	
**Illness duration (Years)**, Mean (SD)	13.2 (9.6)	13.7 (10.5)	0.635
**Age of first contact with DMHs (Years)**, Mean (SD)	25.0 (9.l)	22.8 (8.1)	**0.013**
**BACS scores, Mean (SD)**			
*Verbal memory*	32.6 (11.5)	35.6 (12.2)	**0.015**
*Working memory*	15.6 (4.7)	16.4 (4.8)	0.177
*Token motor task*	57.7 (16.8)	59.3 (17.3)	0.368
*Verbal fluency*	36.6 (12.6)	39.7 (12.9)	**0.021**
*Symbol coding task*	35.4 (12.9)	41.6 (14.1)	**<0.001**
*Tower of London*	14.4 (5.2)	14.9 (5.1)	0.320
**Comorbidity with Personality disorders**			**<0.001**
*No*	152 (70.7)	159 (92.4)	
*Yes*	63 (29.3)	13 (7.6)	
**Lifetime substance use**			0.432
*Never*	50 (22.7)	46 (26.1)	
*Yes*	170 (77.3)	130 (73.9)	
**Present intake of medication**			0.705
*No*	3 (1.4)	4 (2.3)	
*Yes*	218 (98.6)	173 (97.7)	
**Present intake of antipsychotics**			0.191
*No*	3 (1.4)	6 (3.5)	
*Yes*	214 (98.6)	165 (96.5)	

Chi squared or Fisher’s exact test (when *n* < 5 in at least one cell) has been performed for categorical variables; t test has been performed for continuous variables.

Bold entries indicate *p* < 0.05.

### Threshold Disorder and Alignment

**Fig. 1:**
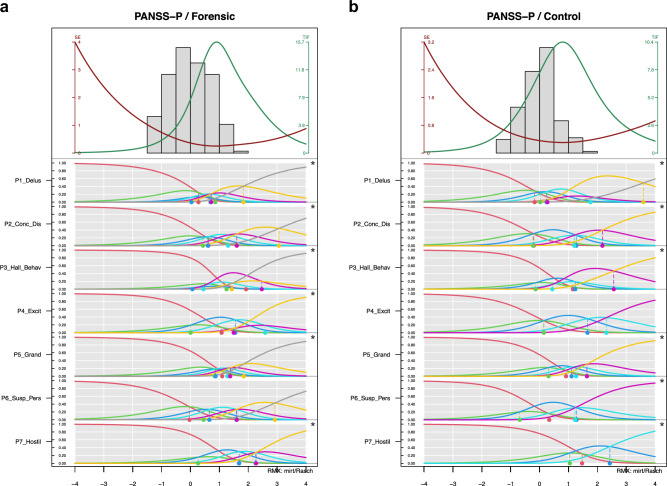
PIccc-Diagrams of the Positive Subscale of the PANSS for the forensic sample (1a/left) and the controlsample (1b/right). **a**, **b** PIccc-Diagrams of the Positive Subscale of the PANSS for the forensic sample (1a/left) and the control sample (1b/right). Notes: The upper part of each diagram shows the person-related information (score histogram; green line: Test Information Curve (TIF); red line: Standard Error (S.E.) of the person parameter estimates); lower part: Category Characteristic Curves of the seven PANSS-P-items showing the probability to choose each response category along the latent scale in the interval –4 to 4. Asterisks on the right border indicate disordered thresholds.

Figure 1 shows the CCCs and the person parameter distributions of the positive subscale in the forensic and the non-forensic samples. Comparing the location of the person parameter distribution in the upper part and the threshold locations in the lower part revealed poor alignment, i.e., the ratings appeared predominantly in the lower part of the latent dimension (approximately –1 to 1), whereas the thresholds were located in the higher regions (approx. 0 to 3). Moreover, all but one item showed disordered threshold, i.e., the categories were not used uniformly as indicated by their labels. Many of them were very close to each other (e.g., item P5), hence the categories appeared too similar to the raters, in spite of the detailed descriptions of their characteristics. Therefore, the CCCs of many categories do not show a unique maximum but “disappear” below other categories. This means that these categories are not necessarily required in the rating process.

**Fig. 2:**
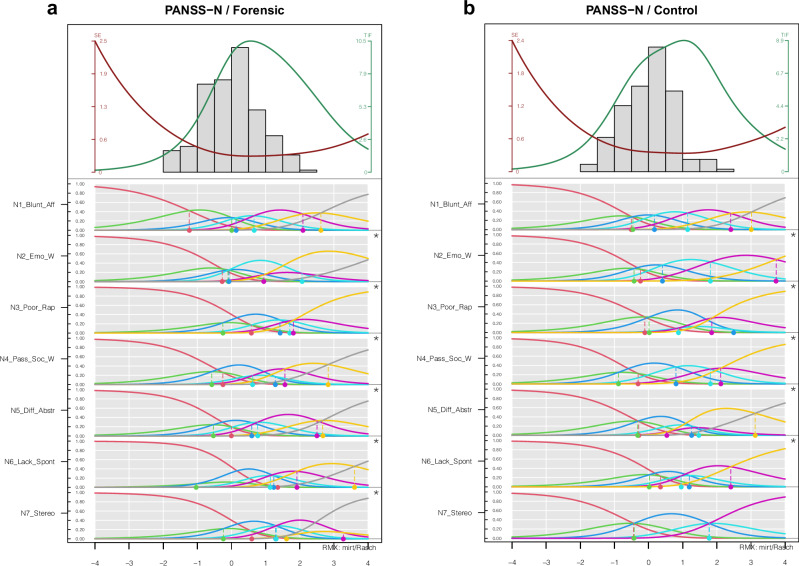
PIccc-Diagrams of the Positive Subscale of the PANSS for the forensic sample (1a/left) and the controlsample (1b/right). **a**, **b** PIccc-Diagrams of the Negative Subscale of the PANSS for the forensic sample (left) and the control sample (right). For Notes see Fig. 1.

**Fig. 3:**
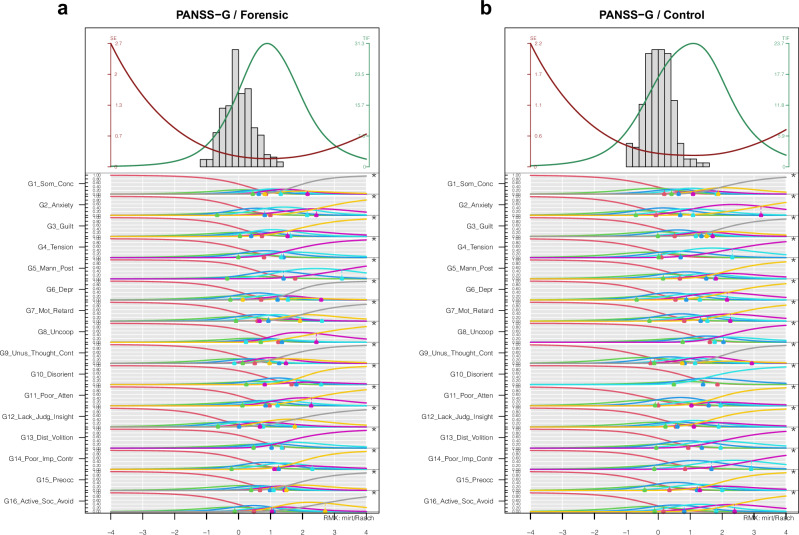
PIccc-Diagrams of the Positive Subscale of the PANSS for the forensic sample (1a/left) and the controlsample (1b/right). **a**, **b** PIccc-Diagrams of the General Subscale of the PANSS for the forensic sample (left) and the control sample (right). For Notes see Fig. 1.

We found disordered thresholds for virtually all items (Figs. 2 and 3). A positive exception was item N1 (Blunted Affect) which showed no disordered threshold in both subsamples (Figs. 2a/b and S2a/b). Also, we found no disordered thresholds for Items P6 (Suspiciousness/Persecution) in the forensic subsample (Fig. 1), P4 (Excitement) in the non-forensic sample (Fig. 1b) and item N7 (Stereotyped Thinking) in the non-forensic sample (Fig. 2b). All items of the General subscale showed disordered thresholds in both samples. All other items exhibited a distorted picture.

### Model Fit

The mean scores of single PANSS items are very similar between both groups (Supplementary Table 1). The score of P3 (Hallucinatory Behavior) was lower in the forensic group while P7 (Hostility) was higher in the control group. None of the negative symptoms differed between the two groups. Scores of G2 (Anxiety), G4 (Tension), G5 (Mannerism and Posturing), G6 (Depression) and G13 (Disturbance of Volition) were lower in the forensic group, while G8 (Uncooperativeness) and G12 (Lack of Judgment and Insight) were higher in the forensic group. The mean scores of PANSS subscales are given in Table 2. The scores for positive symptoms were slightly, but significantly higher in the control group than in the forensic group, while there were no significant differences for the other subscales.

**Table 2:** Mean Scores Of PANSS Subscales.

	Forensic group *N* = 221 Mean (SD)	Control group *N* = 177 Mean (SD)	*p*-value
Positive symptoms	14.8 (6.9)	15.6 (5.7)	**0.020**
Negative Symptoms	18.9 (7.7)	18.3 (6.5)	0.789
General psychopathology	33.9 (11.2)	34.5 (9.2)	0.121
Total score	67.8 (23.0)	68.5 (18.5)	0.226

Bold: *p* < 0.05.

The global M2 test yielded a significant result for all six models thus indicating insufficient fit in both groups (Table 3). Also the RMSEA are larger than the frequently recommended limit of 0.05^32,33^. The Standardized Root Mean Square Residuals (SRMSR) were also larger than their recommended values and the Tucker-Lewis-Index (TLI) and the Comparative Fit Index (CFI) were smaller than the values regarded as sufficient. This called the fit of all models into question.

**Table 3:** Fit Indices Of The Six Models.

	FORENSIC			CONTROL		
	POS	NEG	GEN	POS	NEG	GEN
M2	122.317	125.514	443.841	75.897	132.159	313.254
df	20.000	20.000	119.000	20.000	20.000	119.000
p	0.000	0.000	0.000	0.000	0.000	0.000
NC	6.116	6.276	3.730	3.795	6.608	2.632
RMSEA	0.153	0.156	0.112	0.127	0.180	0.097
RMSEA_5	0.127	0.130	0.101	0.097	0.151	0.084
RMSEA_95	0.179	0.182	0.123	0.158	0.209	0.110
SRMSR	0.131	0.107	0.144	0.115	0.144	0.119
TLI	0.851	0.894	0.848	0.853	0.809	0.802
CFI	0.858	0.899	0.849	0.860	0.818	0.804
AIC	3895.12	4530.544	9064.978	3205.642	3528.262	7281.365
SABIC	3904.144	4539.789	9083.688	3205.392	3527.982	7280.774
HQ	3951.238	4588.032	9181.322	3247.932	3575.678	7381.322
BIC	4034.072	4672.885	9353.049	3309.891	3645.147	7527.771

Notes: POS/NEG/GEN=positive/negative/general subscale of the PANSS.

### Differential Item Functioning

We then assessed whether the items differed with respect to how they were perceived/responded to by forensic and non-forensic subjects. Table 4 shows the item-wise comparisons for both.

**Table 4:** Differential Item Functioning (Dif) In Forensic Patients With Ssd And Controls.

	X^2^	df	*p*
**Positive Subscale**			
P1 Delusions	14.726	6	**0.022**
P2 Conceptual Disorganization	11.402	6	0.077
P3 Hallucinatory Behavior	22.958	6	**0.001**
P4 Excitement	6.953	5	0.224
P5 Grandiosity	3.459	6	0.749
P6 Suspiciousness/Persecution	18.811	6	**0.004**
P7 Hostility	13.397	5	0.**020**
**Negative Subscale**			
N1 Blunted Affect	7.965	6	0.241
N2 Emotional Withdrawal	10.84	6	0.093
N3 Poor Rapport	10.12	5	0.072
N4 Passive/Apathetic Social Withdrawal	11.279	6	0.080
N5 Difficulty in Abstract Thinking	20.501	6	**0.002**
N6 Lack of Spontaneity and flow of Conversation	11.211	6	0.082
N7 Stereotyped Thinking	21.828	6	**0.001**
**General Subscale**			
G1 Somatic Concern	7.195	6	0.303
G2 Anxiety	22.462	5	**0.000**
G3 Guilt feelings	9.902	6	0.129
G4 Tension	12.303	4	**0.015**
G5 Mannerism and Posturing	18.009	5	0.003
G6 Depression	9.69	6	0.138
G7 Motor Retardation	5.624	6	0.467
G8 Uncooperativeness	15.425	5	**0.009**
G9 Unusual Thought Content	21.978	6	**0.001**
G10 Disorientation	4.456	5	0.486
G11 Poor Attention	19.59	5	**0.001**
G12 Lack of Judgment and Insight	103.827	6	**0.000**
G13 Disturbance of Volition	9.144	4	0.058
G14 Poor Impulse Control	3.794	5	0.579
G15 Preoccupation	15.444	6	**0.017**
G16 Active Social Avoidance	6.133	6	0.408

Bold: *p* < 0.05.

A number of the assessed symptoms differed significantly between the two groups: the PANSS Positive subscale showed significant results for Item P1 (Delusions), P3 (Hallucinatory Behavior), P6 (Suspiciousness/Persecution), and P7 (Hostility). In the Negative subscale, Item N5 (Difficulty in abstract thinking) and N7 (Stereotyped thinking) showed significant results. The general psychopathology subscale yielded statistically significant findings for items G2 (Anxiety), G4 (Tension), G5 (Mannerism and Posturing), G8 (Uncooperativeness), G9 (Unusual Thought Content), G11 (Poor Attention), G12 (Lack of Judgment and Insight), and G15 (Preoccupation). Consequently, differences in item response between the two groups of patients with SSD (those who had been violent and those who had not) were of particular significance.

## Discussion

In this study, we compared, for the first time, the psychometric properties of the current PANSS between patients with an SSD who had and had not in the past caused serious interpersonal violence, using the PCM. Our focus was on three key aspects: model fit, disordered thresholds, and DIF.

Several previous studies have applied IRT to the PANSS and revealed significant psychometric concerns. Santor et al. (2007) used non-parametric kernel smoothing and identified 9 out of 30 items as “very good”, while also noting uneven endorsement of response options. Khan et al.^20^ examined data from 7,348 individuals with SSDs using a similar non-parametric approach, and identified 11 “weak” items and concluded to propose a shortened 19-item “Mini-PANSS”. Levine and colleagues^34^ applied a Graded Response Model (GRM) to a five-factor PANSS version and also observed inconsistencies in the functions of response categories and concluded that there was a need for revisions. Anderson et al.^35^ used a bifactor GRM to model both a general factor and specific subscales and concluded that, despite multidimensionality, total PANSS scores remained reliable indicators of symptom severity. Khan et al.^36^ conducted a cross-cultural analysis using the Mantel-Haenszel method to detect DIF and found that although several items showed DIF, only the General Psychopathology subscale warranted further scrutiny. Østergaard and colleagues^37^ applied Rasch models to various PANSS versions and concluded that only a 6-item version—comprising three positive and three negative items—was psychometrically scalable. More recently, Baandrup et al.^38,39^ focused on the Negative subscale using parametric IRT models. They^38^ concluded that the Negative subscale and the total sum scores were invalid and that modifications did not improve its psychometric performance. In their follow-up, Baandrup et al.^39^ identified persistent DIF and found that even dichotomizing the response options failed to resolve these issues. These studies are particularly relevant to our analysis, given their focus on model fit and the structural validity of the PANSS under IRT frameworks.

Our results showed that the PCM did not provide sufficient fit. This finding challenges the widespread practice of using unweighted sum scores as valid measures of symptom severity.

Another critical issue was the over-differentiation of the PANSS response format. The seven-category scale appears to provide more response options than raters can meaningfully discriminate. Many items displayed only two or three functioning categories, indicating that the raters may use the options more in a binary or ternary choice fashion (e.g., yes/no or yes/somewhat/no), rather than using the full range. This issue is characteristic of scales developed in the 1980s, which often adopted extended response formats (i.e., many graded response options) to justify metric-based analyses. However, modern IRT models offer far more appropriate tools for analyzing such ordinal data. Our application of the PCM clearly demonstrated that the seven-category format resulted in inconsistent and heterogeneous utilization, as shown by widespread disordered thresholds and poor model fit. This finding is particularly striking given that each PANSS response category was accompanied by detailed rating guidelines intended to facilitate consistent use. Unfortunately, our results contradict this assumption. Our observations align with previous findings by Levine et al.^34^, who also reported response category problems, and by Khan et al.^19^, who noted the need to better define item response options.

### PANSS in forensic and non-forensic samples

From our perspective, the most novel and important aspect of this study is the comparative analysis between forensic and non-forensic patients with an SSD. To our knowledge, this is the first investigation to examine PANSS item functioning using IRT and DIF analysis in a forensic psychiatric population. DIF analyses revealed significant item-level differences between the two groups, suggesting that violent individuals in forensic settings interpret and respond to PANSS items differently than those non-violent subjects in general psychiatric care.

Among the positive symptom items, the greatest differences were found in Item P1 (Delusions), P3 (Hallucinatory Behavior), P6 (Suspiciousness/Persecution), and P7 (Hostility). These items have well-recognized clinical relevance, as they reflect dynamic risk factors for violence often found on mental state examination. Previous studies have demonstrated that hallucinations accompanied by delusional interpretations, and delusional beliefs involving persecution or suspiciousness, are associated with the risk of serious violence in people with schizophrenia^8,14,40^. Similarly, the PANSS Hostility item may reflect delusions, which have been linked to violent behavior, particularly during the first episode of psychosis^16^. Hallucinatory behavior associated with command hallucinations has also been identified as a key dynamic risk factor in the perpetration of violence^15^.

For the negative subscale, Items N5 (Difficulty in Abstract Thinking) and N7 (Stereotyped Thinking) showed significant DIF. These items are particularly relevant given their reported association with violence in a meta-analysis by Reinharth et al.^41^. Similarly, Ntounas and colleagues^7^ found that dangerousness among patients with paranoid schizophrenia was associated with difficulties in abstract thinking and stereotyped thinking. Witt et al.^9^ who performed a meta-regression reported that higher positive symptom scores predicted violence while negative symptom scores did not.

For the General Psychopathology subscale, the greatest differences were found in Item G2 (Anxiety), G4 (Tension), G8 (Uncooperativeness), G9 (Unusual Thought Content), G11 (Poor Attention), G12 (Lack of Judgement and Insight), and G15 (Preoccupation). Unusual thought content and preoccupation are often associated with delusions, while anxiety and tension are often consequences of psychotic symptoms^14^. Persons with marked hallucinations often suffer from poor attention^14^. Lack of insight and uncooperativeness often result in poor medication compliance increasing the risk of relapses. Further, lack of insight has been consistently associated with violence risk in systematic reviews and meta-regression analyses^9^.

These DIF results likely reflect the markedly different histories and treatment contexts between the two populations. While general psychiatric patients often seek help voluntarily due to symptom severity or distress, forensic patients typically lack insight, are harder to engage, and often receive involuntary treatment. Moreover, forensic psychiatrists recognize their dual roles, acting both as treating psychiatrists but also as part of the systems that assure public safety and advised the courts. Consequently, forensic patients with an SSD may be more reluctant to seek out help, explore the nature of their symptoms, and may be motivated to minimize or conceal their symptoms.

The forensic group showed a lower score on the Positive symptom subscale, but more often comorbid personality disorders than the control group. Further, the groups differed in several cognitive domains according to BACS. We don’t know whether this could contribute to interviewers’ PANSS ratings among forensic subjects differently than those of non-violent subjects. Further clinical aspect such as duration of untreated psychosis, dosage of antipsychotics, or stability of psychopathology (which was not assessed in our study) might influence the psychometric disparities which were found in the present study. In addition, aspects such as self-stigma or practitioners’ attitudes to forensic patients might influence psychometric aspects^42–44^. Future studies should consider such aspects which need more complex analyses, which, in turn, require larger samples. Nevertheless, this is the first study comparing the psychometric properties of PANSS between patients with an SSD using PCM.

### Limitations

This study has some limitations that potentially reduce its generalizability. Firstly about 30% of both samples refused to participate. Since we did not collect any data on those who did not participate, we do not know if those who refused differed in psychopathology or other characteristics from those who participated in this study.

Despite the fact that we planned the samples to be sex-balanced, the sex distribution of the two samples was imbalanced due to problems in recruitment linked to the COVID-19 pandemic.

## Conclusions

This study represents the first application of IRT and DIF analyses to the PANSS in a forensic psychiatric sample of patients with SSDs. Our findings reveal significant psychometric disparities between the forensic and non-forensic populations. Those differences likely reflect variations in treatment setting, assessment context, and patient motivation. While individual PANSS items provided useful information about specific symptoms, our results call into question the validity of the PANSS total and subscale scores, particularly in forensic contexts.

Given these findings and the widespread use of the PANSS in both clinical and forensic settings, there is an urgent need for scale revision or adaptation. The present study highlights serious limitations in the PANSS scoring methodology, and neither population demonstrated psychometric support for the use of sum scores. Further research using larger samples in both clinical and forensic populations is essential to confirm and expand upon these findings and to support the development of more reliable and valid assessment tools.

### Ethical standards

The authors assert that all procedures contributing to this work comply with the ethical standards of the relevant national and institutional committees on human experimentation and with the Helsinki Declaration of 1975, as revised in 2008.

## Supplementary information

Supplemental file

## Data Availability

The project will fully embrace the open access data policy of H2020 to make data FAIR (Findable, Accessible, Interoperable, and Re-usable), and all data gathered in the framework of the project are stored in a public repository (10.5281/zenodo.4442372) accessible to all scientists willing to carry out additional analyses.
