# Supplementary material for: Evaluating the PANSS using item response theory in forensic psychiatric samples from five European nations
Source: Schizophrenia (Heidelb). 2025 Nov 25;11(1):141. doi: 10.1038/s41537-025-00668-0 (PMC12647602; doi:10.1038/s41537-025-00668-0)
Supplement: Supplementary file 1 — Supplemental file [file 41537_2025_668_MOESM1_ESM.pdf]

Supplement to

Evaluating the PANSS Using Item Response Theory in Forensic Psychiatric Samples from Five European Nations.

Andreas Wippel[1], *Giovanni de Girolamo*[2], *Pawel Gosek*[3], *Janusz Heitzman*[4], *Laura Iozzino*[2], *Inga Markiewicz*[4], *Donato Martella*[2], *Marco Picchioni*[5,6], *Hans-Joachim Salize*[7], *Annemarie Unger*[1], *Johannes Wancata*[1], *Rainer W. Alexandrowicz*[8]

1. Medical University of Vienna, Clinical Division of Social Psychiatry, Vienna, Austria
2. IRCCS Istituto Centro San Giovanni di Dio Fatebenefratelli, Unit of Epidemiological Psychiatry and Digital Mental Health, Brescia, Italy
3. Department of Psychiatry, Center of Postgraduate Medical Education, Warsaw
4. Institute of Psychiatry and Neurology, Department of Forensic Psychiatry, Warsaw, Poland
5. Department of Forensic and Neurodevelopmental Science, Institute of Psychiatry, Psychology and Neuroscience, King's College London, UK
6. St Magnus Hospital, Haslemere, Surrey, UK
7. Central Institute of Mental Health Mannheim, Medical Faculty Mannheim/Heidelberg University, Heidelberg Germany
8. University of Klagenfurt, Institute of Psychology, Klagenfurt, Austria

Abstract

Item Response Theory (IRT) describes a set of statistical models describing how individual items in a test or questionnaire relate to the underling characteristic or trait that the test claims to measure. Until now IRT models have not been applied to the Positive and Negative Syndrome Scale (PANSS) in forensic and general psychiatric samples to establish its psychometric properties and explore the link between psychotic symptom severity and violent behavior in schizophrenia. This study investigated patients with schizophrenia spectrum disorders and a history of violence from forensic institutions and non-violent patients from general psychiatric settings in five European countries. A total of 398 participants were assessed using the PANSS. IRT analysis revealed a poor model fit for the Partial Credit Model (PCM) with considerably disordered thresholds for most items. Differential item functioning (DIF) revealed significant differences between the two groups, notably for items hypothetically linked to violence risk, such as delusions and hostility. These findings reveal potential limitations when trying to compare PANSS scores across these two clinical populations.

Keywords

Schizophrenia, Positive and Negative Syndrome Scale, symptoms severity, Item Response Theory

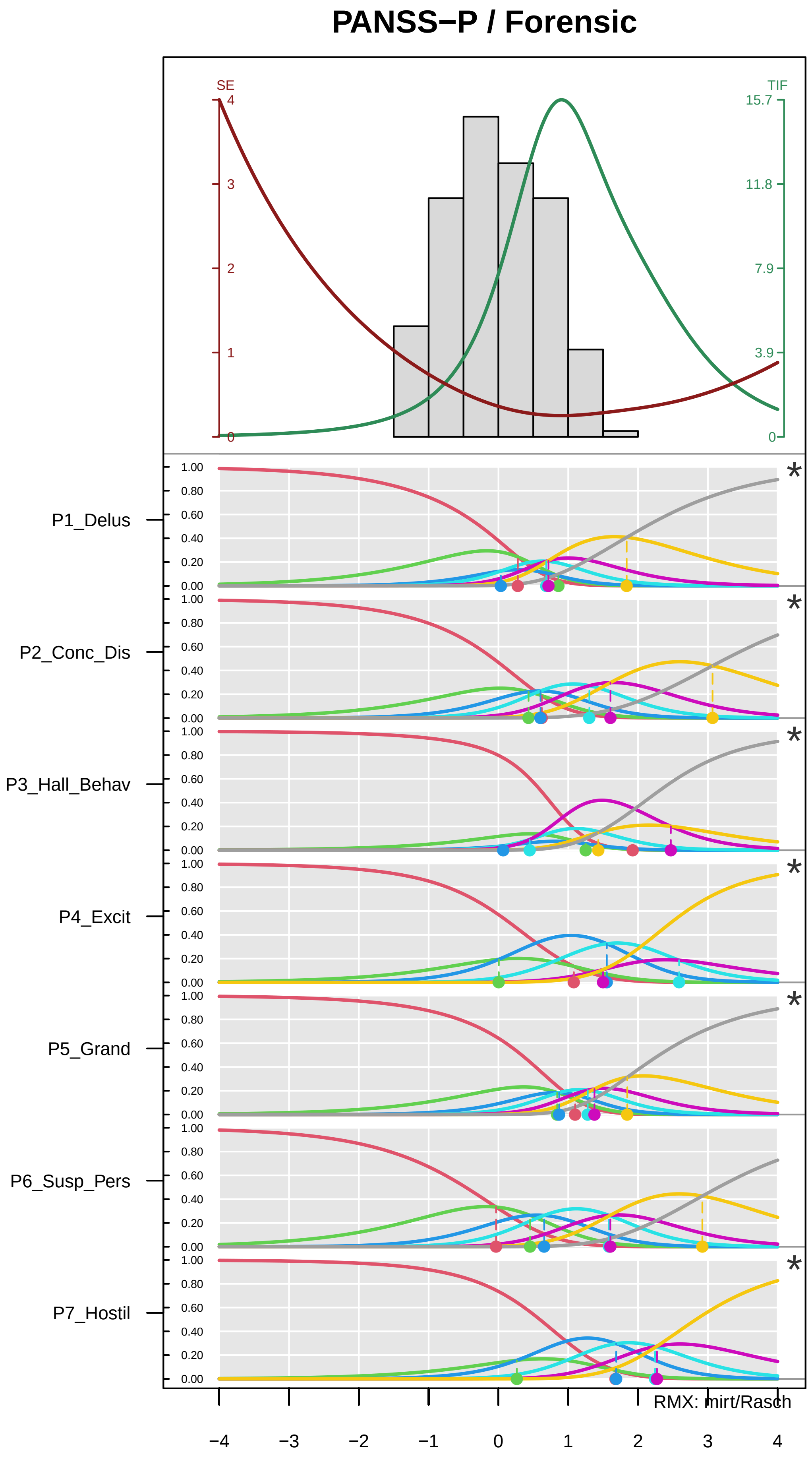
PANSS−P / Forensic
SE
TIF
4
3
2
1
0
15.7
11.8
7.9
3.9
0
1.00
0.80
0.60
0.40
0.20
0.00
P1_Delus
P2_Conc_Dis
P3_Hall_Behav
P4_Excit
P5_Grand
P6_Susp_Pers
P7_Hostil
*
RMX: mirt/Rasch
−4
−3
−2
−1
0
1
2
3
4

Figure S1a

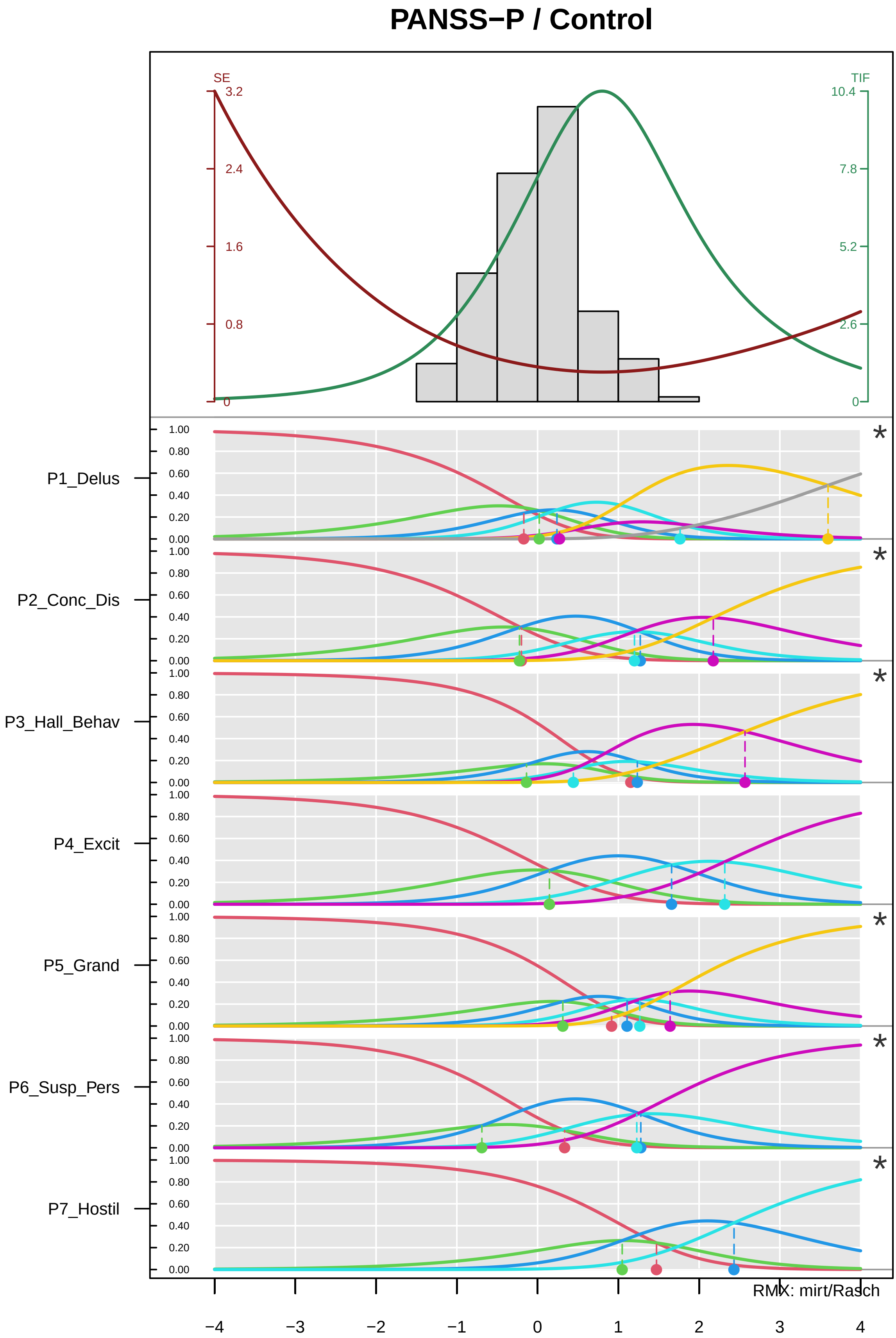

PANSS−P / Control
SE
3.2
2.4
1.6
0.8
0
TIF
10.4
7.8
5.2
2.6
0
P1_Delus
P2_Conc_Dis
P3_Hall_Behav
P4_Excit
P5_Grand
P6_Susp_Pers
P7_Hostil
1.00
0.80
0.60
0.40
0.20
0.00
RMX: mirt/Rasch
−4
−3
−2
−1
0
1
2
3
4

Figure S1b

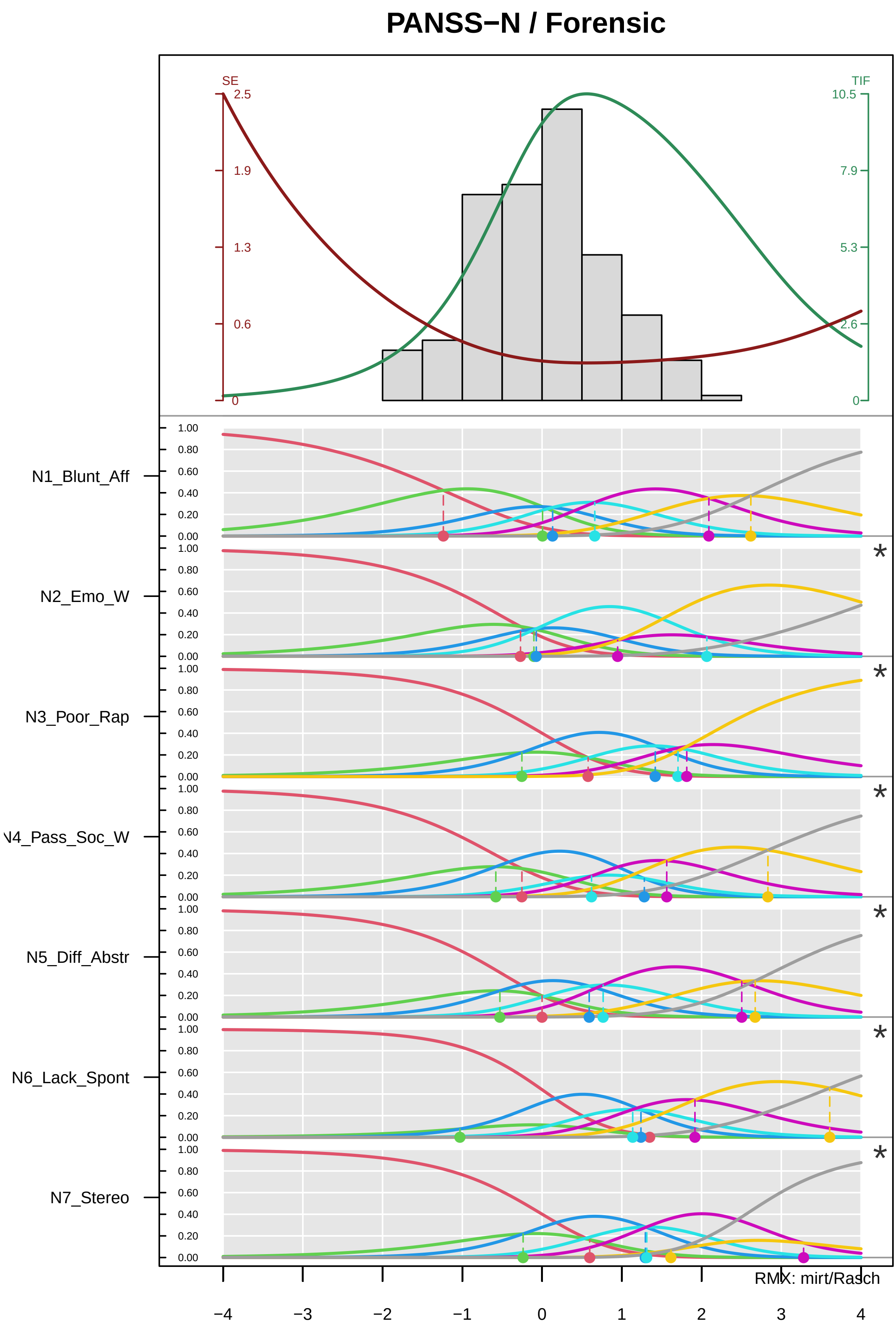

PANSS−N / Forensic
SE
2.5
1.9
1.3
0.6
0
TIF
10.5
7.9
5.3
2.6
0
N1_Blunt_Aff
N2_Emo_W
N3_Poor_Rap
N4_Pass_Soc_W
N5_Diff_Abstr
N6_Lack_Spont
N7_Stereo
1.00
0.80
0.60
0.40
0.20
0.00
RMX: mirt/Rasch
−4
−3
−2
−1
0
1
2
3
4

Figure S2a

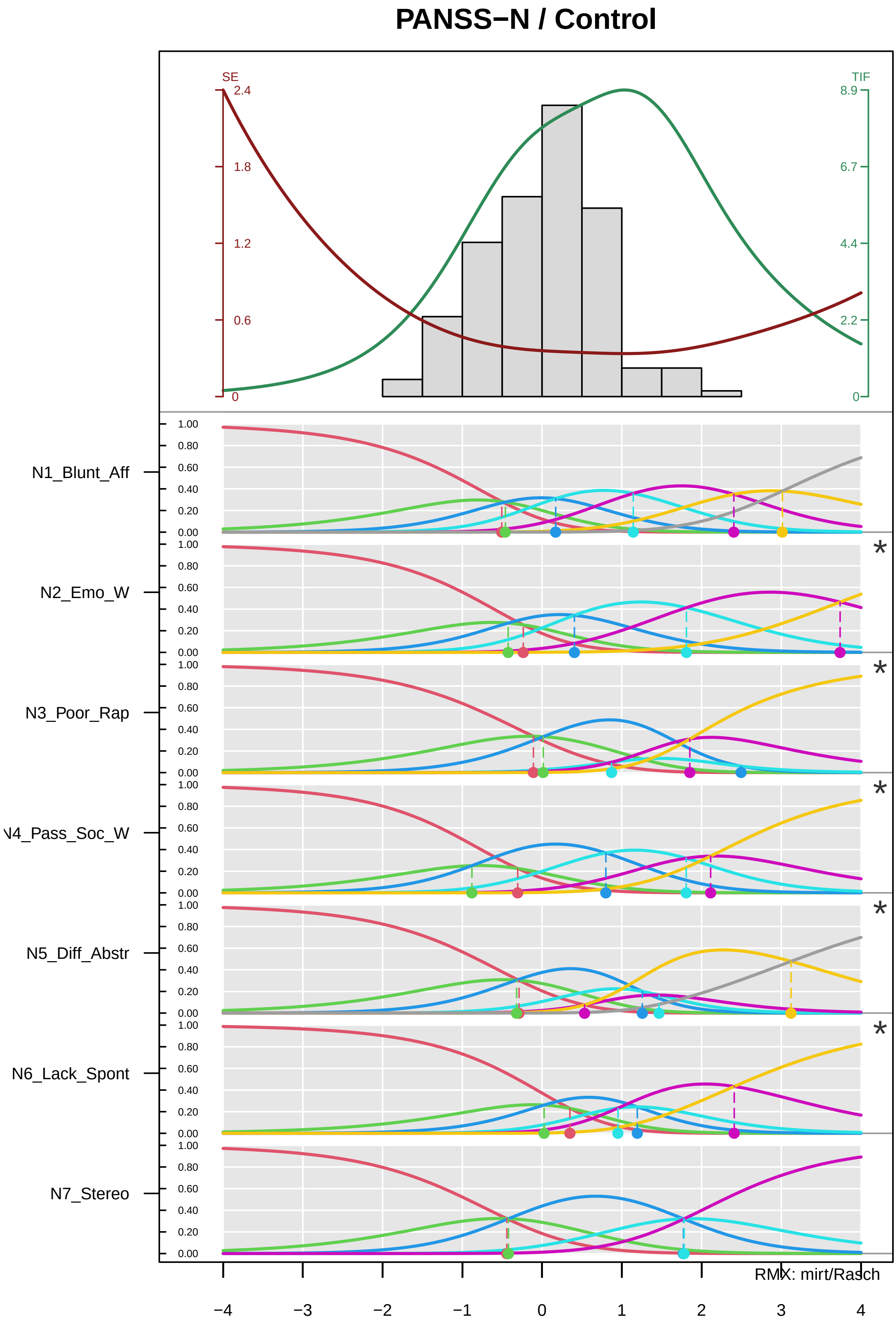
PANSS−N / Control
SE
2.4
1.8
1.2
0.6
0
TIF
8.9
6.7
4.4
2.2
0
N1_Blunt_Aff
N2_Emo_W
N3_Poor_Rap
N4_Pass_Soc_W
N5_Diff_Abstr
N6_Lack_Spont
N7_Stereo
1.00
0.80
0.60
0.40
0.20
0.00
*
RMX: mirt/Rasch
−4
−3
−2
−1
0
1
2
3
4

Figure S2b

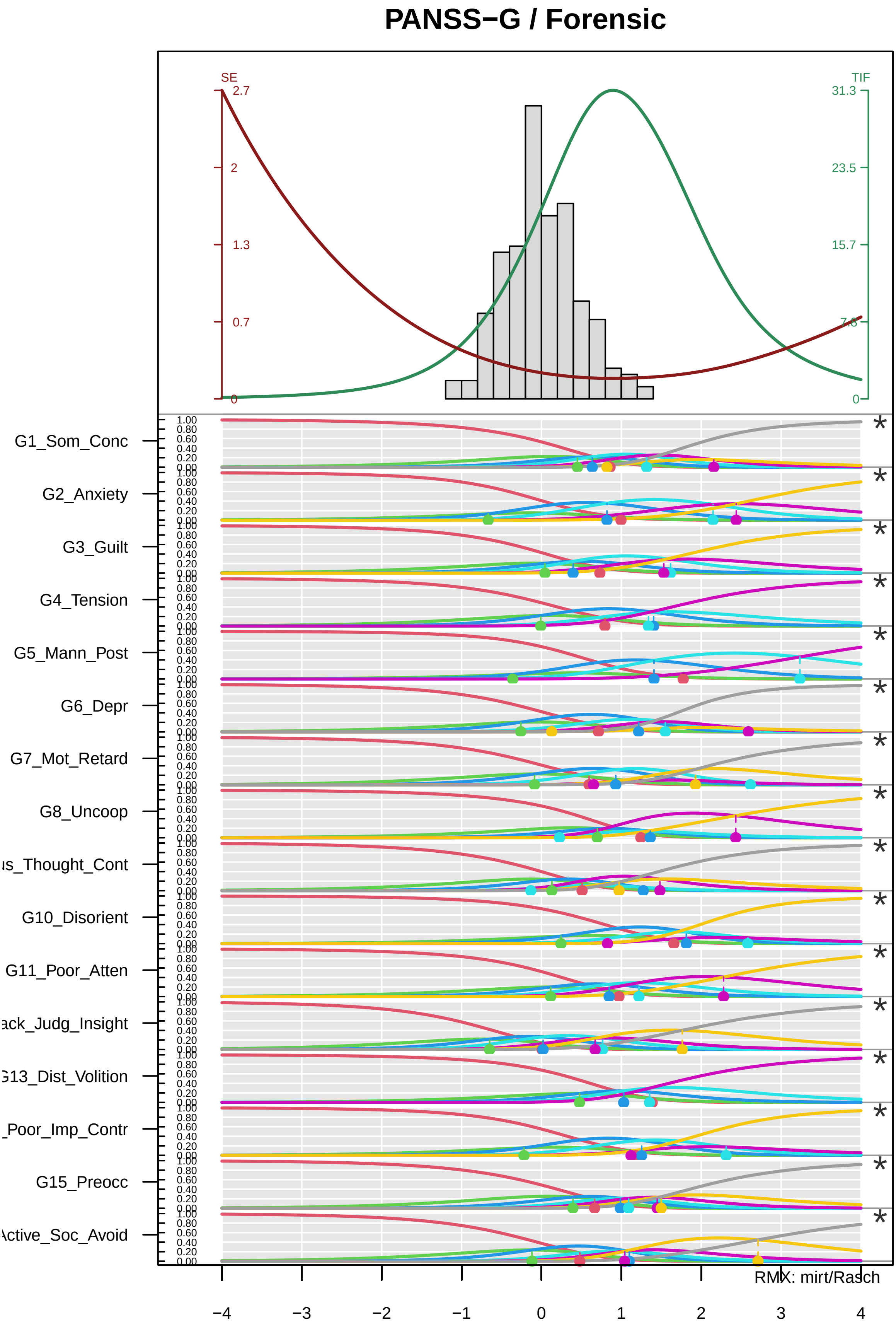
PANSS−G / Forensic
SE
TIF
G1_Som_Conc
G2_Anxiety
G3_Guilt
G4_Tension
G5_Mann_Post
G6_Depr
G7_Mot_Retard
G8_Uncoop
G10_Disorient
G11_Poor_Atten
G15_Preocc
RMX: mirt/Rasch

Figure S3a

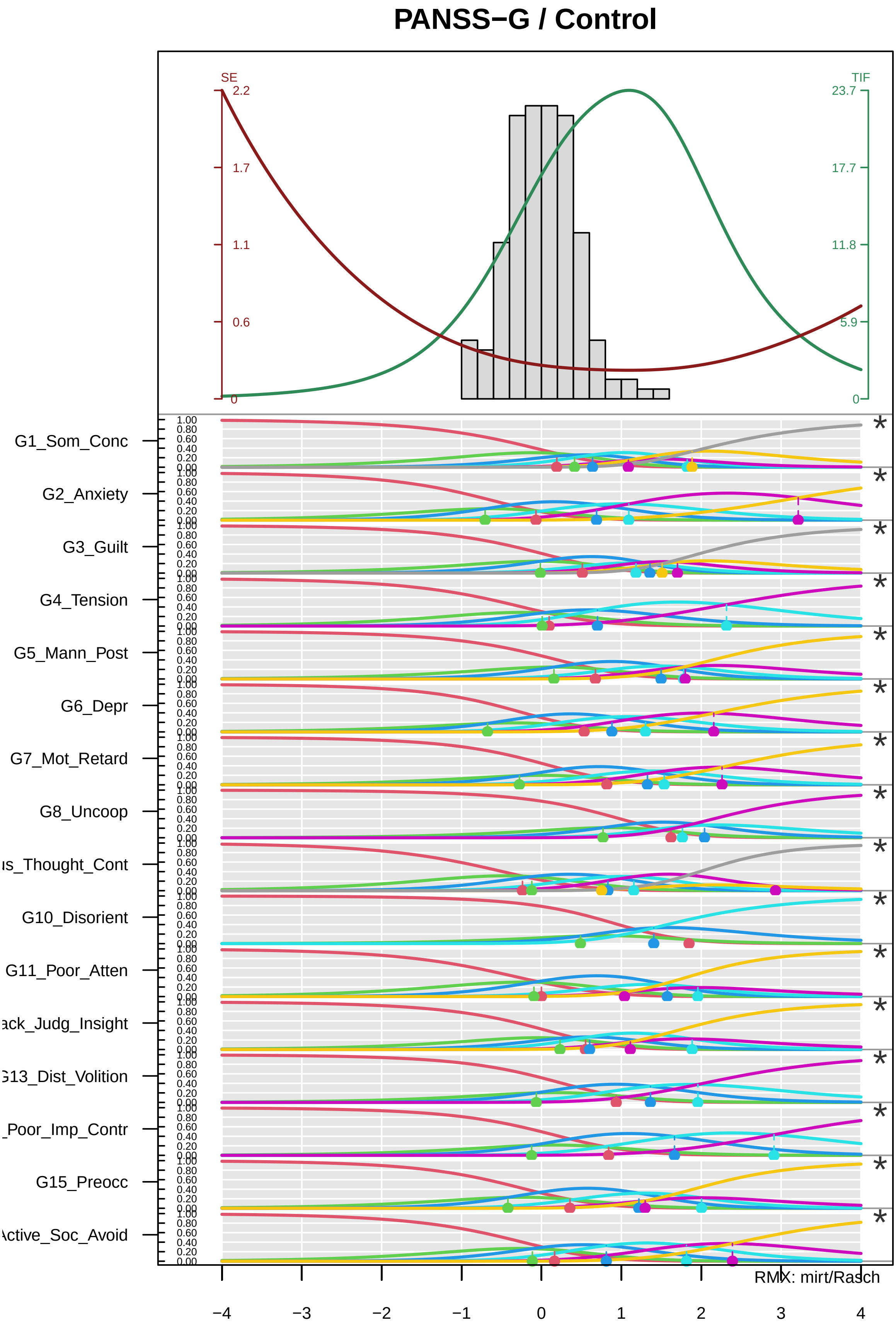
PANSS−G / Control
SE
2.2
1.7
1.1
0.6
0
TIF
23.7
17.7
11.8
5.9
0
G1_Som_Conc
G2_Anxiety
G3_Guilt
G4_Tension
G5_Mann_Post
G6_Depr
G7_Mot_Retard
G8_Uncoop
ıs_Thought_Cont
G10_Disorient
G11_Poor_Atten
ack_Judg_Insight
G13_Dist_Volition
_Poor_Imp_Contr
G15_Preocc
Active_Soc_Avoid
RMX: mirt/Rasch
−4
−3
−2
−1
0
1
2
3
4

Figure S3b

Table S1: MEAN SCORES OF SINGLE PANSS ITEMS (SD = STANDARD DEVIATION)

	**Forensic group**	**Control group**	*t*-**Test**		
	**Mean (SD)**	**Mean (SD)**	*t*	*df*	2-sided *p*
**Positive Subscale**					
P1 Delusions	2.76 (1.89)	2.83 (1.57)	0.391	389	0.696
P2 Conceptual Disorganization	2.27 (1.48)	2.53 (1.27)	1.779	390	0.076
P3 Hallucinatory Behavior	1.85 (1.53)	2.17 (1.44)	2.103	389	0.036
P4 Excitement	1.90 (1.15)	2.10 (1.05)	1.773	391	0.077
P5 Grandiosity	1.95 (1.45)	2.03 (1.34)	0.563	390	0.574
P6 Suspiciousness/Persecution	2.41 (1.40)	2.49 (1.25)	0.571	389	0.569
P7 Hostility	1.63 (1.03)	1.41 (0.73)	–2.348	391	0.019
**Negative Subscale**					
N1 Blunted Affect	3.20 (1.52)	3.04 1.43)	–1.039	391	0.299
N2 Emotional Withdrawal	2.90 (1.52)	2.74 (1.29)	–1.105	388	0.270
N3 Poor Rapport	2.28 (1.36)	2.26 (1.21)	–0.143	391	0.886
N4 Passive/Apathetic Social Withdrawal	2.88 (1.56)	2.83 (1.27)	–0.334	390	0.738
N5 Difficulty in Abstract Thinking	2.89 (1.54)	2.73 (1.51)	–1.072	390	0.284
N6 Lack of Spontaneity and Flow of Conversation	2.42 (1.54)	2.36 (1.40)	–0.455	391	0.649
N7 Stereotyped Thinking	2.32 (1.40)	2.46 (1.08)	1.079	389	0.281
**General Subscale**					
G1 Somatic Concern	2.02 (1.34)	2.25 (1.30)	1.698	388	0.090
G2 Anxiety	2.26 (1.25)	2.80 (1.25)	4.309	389	<0.001
G3 Guilt feelings	2.27 (1.36)	2.17 (1.27)	–0.733	384	0.464
G4 Tension	1.98 (1.13)	2.32 (1.15)	2.903	390	0.004
G5 Mannerism and Posturing	1.68 (1.03)	1.94 (1.09)	2.400	390	0.017
G6 Depression	2.18 (1.27)	2.51 (1.30)	2.531	390	0.012
G7 Motor Retardation	2.17 (1.25)	2.09 (1.18)	–0.705	391	0.481
G8 Uncooperativeness	1.73 (1.18)	1.40 (0.77)	–3.217	391	0.001
G9 Unusual Thought Content	2.48 (1.66)	2.57 (1.26)	0.610	388	0.542
G10 Disorientation	1.53 (0.92)	1.41 (0.80)	–1.328	391	0.185
G11 Poor Attention	2.03 (1.27)	2.26 (1.11)	1.894	391	0.059
G12 Lack of Judgment and Insight	3.35 (1.65)	2.17 (1.28)	–7.788	390	<0.001
G13 Disturbance of Volition	1.67 (1.07)	1.91 (1.09)	2.221	391	0.027
G14 Poor Impulse Control	1.92 (1.16)	1.90 (1.02)	–0.139	391	0.890
G15 Preoccupation	2.08 (1.36)	2.33 (1.18)	1.885	391	0.060
G16 Active Social Avoidance	2.35 (1.40)	2.36 (1.20)	0.116	389	0.908
